# Impact of oral condition on the quality of life of homeless people

**DOI:** 10.11606/S1518-8787.2019053000718

**Published:** 2019-02-20

**Authors:** Juliana Aparecida de Campos Lawder, Marcos André de Matos, João Batista de Souza, Maria do Carmo Matias Freire

**Affiliations:** I Universidade Federal de Goiás. Faculdade de Odontologia. Programa de Pós-Graduação em Odontologia. Goiânia, GO, Brasil; II Universidade Federal de Goiás. Faculdade de Enfermagem. Programa de Pós-Graduação em Enfermagem. Goiânia, GO, Brasil

**Keywords:** Homeless Persons, Oral Health, Sickness Impact Profile, Health Vulnerability, Quality of Life, Pessoas em Situação de Rua, Saúde Bucal, Perfil de Impacto da Doença, Vulnerabilidade em Saúde, Qualidade de Vida

## Abstract

**OBJECTIVE:**

To describe the prevalence of oral health impact on quality of life and its association with the dental condition and sociodemographic factors of homeless people.

**METHODS:**

The sample was composed of 116 adults, temporarily attended by a public institution in the municipality of Goiânia, state of Goiás. Interviews were carried out including the Oral Impact on Daily Performance instrument and sociodemographic aspects. Clinical examinations were done by a trained examiner considering criteria of the World Health Organization. We evaluated dental caries (DMFT index) and use or need to use some type of prosthesis. For the statistical analysis of data, we used Pearson's Chi-square and Fisher's exact tests and Poisson regression with robust variance.

**RESULTS:**

Of the total respondents, 81.9% had at least one daily performance affected by dental problems in the six months prior the survey. The most prevalent dental conditions were: need for lower arch (76.7%) and upper arch prosthesis (69.0%); untreated caries (75.9%); and high DMFT (57.8%). In bivariate analysis, only the need for upper prosthesis variable was associated with the impact (high Oral Impact on Daily Performance). In the regression model, adjusted for time in the institution, age, and sex, this association remained significant (p = 0.015). Individuals without need for upper prosthesis had prevalence of high impact on daily performance 55% lower than those in need of this type of prosthesis (p = 0.018).

**CONCLUSIONS:**

The prevalence of oral health impact on quality of life of homeless people was high and higher than that verified in the overall Brazilian population. The impact was associated with the need for upper prosthesis, regardless of sociodemographic characteristics of the individuals.

## RESUMO

**OBJETIVO:**

Descrever a prevalência do impacto da saúde bucal na qualidade de vida e sua associação com a condição dentária e fatores sociodemográficos de indivíduos em situação de rua.

**MÉTODOS:**

A amostra foi composta por 116 indivíduos adultos, atendidos temporariamente por uma instituição pública no município de Goiânia, GO. Foram realizadas entrevistas incluindo o instrumento Impacto Bucal no Desempenho Diário e aspectos sociodemográficos. Exames clínicos foram feitos por uma examinadora calibrada, utilizando critérios da Organização Mundial de Saúde. Foram avaliados cárie dentária (índice CPOD) e uso ou necessidade de uso de algum tipo de prótese. Na análise estatística dos dados, usamos os testes qui-quadrado de Pearson e exato de Fisher e a regressão de Poisson com variância robusta.

**RESULTADOS:**

Do total de participantes, 81,9% tiveram pelo menos um desempenho diário afetado por problemas odontológicos nos seis meses anteriores à pesquisa. As condições dentárias mais prevalentes foram: necessidade de prótese na arcada inferior (76,7%) e na superior (69,0%); cárie não tratada (75,9%); e CPOD alto (57,8%). Na análise bivariada, apenas a variável necessidade de prótese superior foi associada ao impacto (Impacto Bucal no Desempenho Diário alto). No modelo de regressão, ajustando-se por tempo na instituição, idade e sexo, essa associação se manteve significativa (p = 0,015). Os indivíduos sem necessidade de prótese superior tiveram prevalência de alto impacto no desempenho diário 55% menor do que aqueles com necessidade desse tipo de prótese (p = 0,018).

**CONCLUSÕES:**

A prevalência de impacto da saúde bucal na qualidade de vida dos indivíduos em situação de rua foi alta e superior à verificada na população brasileira em geral. O impacto foi associado à necessidade de prótese superior, independente das características sociodemográficas dos indivíduos.

## INTRODUCTION

Oral problems, particularly dental caries and its consequences, have high prevalence in the population and are related to pain, suffering, mutilation and privations^1^.

Studies that assess the perception of individuals on their oral conditions are an important contribution to the understanding of the social impact of diseases and other health outcomes^2^. Moreover, they may be relevant to support public policies seeking to establish priorities and reduce inequities and the impact of outcomes on people's quality of life. In this sense, several indicators aiming to integrate subjective measures into clinical data have been developed and validated^3^. The use of these indicators in the field of dental public health has been growing, but studies on vulnerable social groups are still incipient, in particular those on homeless people.

In Brazil, there are no conclusive studies on the profile and the number of people living in the streets; however, it is a growing, heterogeneous, and fluctuating social group, with difficulties of access to healthcare services^4^. Since they are socially excluded, they end up not being focus of healthcare initiatives and scientific research. Accordingly, strategies and actions aimed at improving healthcare initiatives for the population of homeless people have been proposed by the Brazilian Ministry of Health in the last years^5^ ^,^ ^6^.

In addition to the low priority given to this population, having no fixed location of permanence, the insecurity and the adverse psychic and physical conditions of these individuals are factors that hinder investigations comprising all methodological rigor advocated in the epidemiology field. The unique national survey on homeless people did not address specific questions about oral health, and this was not cited by the participants when reporting their major health problems^4^. However, for a group of homeless people interviewed in a qualitative research conducted in Salvador, state of Bahia, dental problems were among the major health problems faced^7^.

Studies on the impact of oral health on the quality of life of homeless people were conducted in Australia^8^, United States of America^9^, China^10^, and European countries^11^ ^–^ ^13^. Results demonstrated a precarious condition of oral health, with indexes higher than those verified in the general population, and which negatively impact on the quality of life of these individuals.

In Brazil, studies on the oral health of homeless people are scarce. A single study evaluated its impact on the quality of life, using descriptive approach and restricted to men of a municipality in the Southeast region, and showed high prevalence of self-reported morbidity and negative impact^14^.

Further investigations are necessary to elucidate the extent to which oral health negatively affects the life of homeless people in Brazil, which clinical conditions influence this impact, and which are other associated factors. Based on the unfavorable-life context of these individuals, we assume that caries and consequent edentulism negatively affect their daily performance. The findings may contribute to develop public policies aimed at this socially marginalized group, but which has the right to oral health with quality of life.

Our study aimed to describe the prevalence of oral health impact on quality of life and its association with the dental condition and sociodemographic factors of homeless people.

## METHODS

Cross-sectional study, conducted at *Casa de Passagem* (Temporary Shelter), the only municipal public institution that temporarily houses homeless people in the municipality of Goiânia, state of Goiás, Brazil. This research is part of a wider project entitled “Avaliação da situação de saúde da população em situação de rua de Goiânia-GO, Brasil Central: elementos para o cuidado a grupos sociais vulneráveis,” under the coordination of Faculdade de Enfermagem – Universidade Federal de Goiás.

For the investigation of oral-health-related aspects, the study population was composed of a convenience sample of 120 individuals. Inclusion criteria were: being at the institution during the period of data collection (three days of the week alternating between September 2014 and August 2015) and aging 18 years or older. Those who were under the influence of substances that put the researchers’ integrity at risk were excluded.

Prior to the completion of the survey, a pilot study was developed. Data was collected by an interview conducted at the *Casa de Passagem* and by oral clinical examination. Interviewers (20 students and healthcare professionals) were previously trained. The instrument featured questions related to sociodemographic characteristics of individuals and a block of questions on the impact of oral condition on the daily performance of activities, measured by the Oral Impact on Daily Performance (OIDP)^15^.

For the OIDP, individuals were questioned in relation to the last six months about a problem with their oral health that caused difficulties or detriments in the following performances: eating; talking; brushing teeth; sleeping and relaxing; smiling or speaking without shame; maintaining a balanced emotional state without getting nervous or angry; performing the work or main activity; and having contact with people. Following the methodology of the national oral health research (*Projeto SBBrasil 2010* [2010 SBBrasil Project])^16^, the possible responses to each of the eight items were: yes; no; do not know; or did not answer.

Sociodemographic variables and their categories were: age; sex; self-reported skin color/race; common-law relationship: lives with a partner (yes, no); education level, measured in years of study: high (nine or over) and low (up to eight); period living in the streets; and length of stay at the institution. The classification of skin color/race was based on the categories of the Brazilian Institute of Geography and Statistics (IBGE)^17^: whites, blacks, *pardos*, yellows, and indigenous. For statistical analysis, these categories were dichotomized (white, non-white).

The following dental conditions were evaluated: dental caries and the use and need of prostheses in both arches. Caries was evaluated by the DMFT index (decayed, missing, filled teeth), using the criteria of the World Health Organization (WHO)^18^. For the assessment of the use and the need for dental prosthesis the following types of prostheses were considered: fixed denture, removable partial denture, and complete denture. The prevalence was determined by the presence or need for at least one type of dental prosthesis in each of the upper and lower arches.

Clinical data were collected by an examiner (dental surgeon) who was trained before conducting the survey. For estimation of the intra-examiner agreement, Kappa statistic was used, with values of 0.97 (almost perfect agreement) for caries and 1.0 (100% agreement) for the need and use of prosthesis. The periodontal probe, recommended by the WHO, and a mouth mirror were used for oral examination.

Statistical analysis initially comprised the descriptive analysis of the variables. The dependent variable was the oral health impact on quality of life (OIDP) and independent variables were sociodemographic characteristics and the dental condition of individuals. Initially, the prevalence of each item of the OIDP was estimated considering the answers: yes (presence) and no (absence). To estimate the prevalence of total impact (OIDP), the presence of at least one item with impact was considered, being dichotomized in “yes” (sum of the eight items totaling a value equal to or more than one) and “no” (sum of the eight items totaling zero).

Next, bivariate analyses were performed to verify the association between impact and each of the other sociodemographic and clinical variables, using the Pearson's Chi-square and Fisher's exact tests. For the analysis of each of the items of the OIPD separately, the presence or absence of impact were considered. Since the prevalence of total impact was low, we decided to categorize it into two levels, based on the median: low impact (OIDP from 0 to 3) and high impact (OIDP from 4 to 8).

The clinical variable that was associated with the impact (total OIDP), with p < 0.05 significance level, was analyzed by a Poisson regression with robust variance model. The selection of possible confounding variables in each model was based on the value of p < 0.20 obtained in the bivariate analyses. We kept in the final model only those with p < 0.05 significance. Demographic variables (age and sex) were inserted into the adjusted model regardless of the p value. Estimations were performed using the Statistical Package for the Social Sciences (SPSS) program for Windows, version 17.0.

The project was approved by the Research Ethics Committee of the Universidade Federal de Goiás (Protocol 045/2013). Individuals invited to participate were guided on the research and those who agreed to participate signed an informed consent form.

## RESULTS

Of 120 individuals invited to participate in the study, 116 have accepted (response rate = 96.7%). Sociodemographic characteristics of the participants are presented in Table 1. The sample was predominantly composed of men (84.5%) and the age ranged from 18 to 77 years (mean = 38.7). As for the self-reported skin color/race, more than half were pardos (51.7%). Most did not live with a partner (82.7%), had low education level (65.5%), and were more than 14 days in the institution (56.9%). Approximately 40% of individuals reported to be less than a year living in the streets, and 56.9% were in the institution for at least 14 days.

**Table 1:** Sociodemographic characteristics and oral condition of homeless people. Goiânia, state of Goiás, Brazil. (n = 116)

Variable		n	%
Age (n = 115)	18 to 35 years	56	48.3
	36 to 77 years	59	50.8
Sex	Male	98	84.5
	Female	18	15.5
Skin color (n = 113)	White	26	22.4
	Black	21	18.1
	Mixed-race	60	51.7
	Yellow	2	1.7
	Indigenous	4	3.4
Lives with a partner (n = 113)	Yes	17	14.7
	No	96	82.7
Education level	High (9 years or over)	36	31.0
	Low (up to 8 years of study)	76	65.6
Time living on the street (n = 78)	Up to 1 year	46	39.7
	More than 1 year	32	27.5
Length of stay in the institution (n = 113)	1 to 13 days	47	40.5
	14 days or over	66	56.9
DMFT	Low	49	42.2
	High	67	57.8
Decayed teeth	No	28	24.1
	Yes	88	75.9
Missing teeth	No	10	8.6
	Yes	106	91.4
Using upper prosthesis	No	100	86.2
	Yes	16	13.8
Using lower prosthesis	No	113	97.4
	Yes	3	2.6
Need for upper prosthesis	No	36	31.0
	Yes	80	69.0
Need for lower prosthesis	No	27	23.3
	Yes	89	76.7

Regarding the dental condition, only two individuals had no caries (1.7%). The mean DMFT was 14.41 (SD = 9.14), composed by 59.8% of missing, 29.4% decayed, and 10.9% filled teeth. Most of the individuals had high DMFT (58.0%) and teeth with untreated caries (75.9%) (Table 1). The use of prosthesis was identified in 13.8% of the individuals in the upper arch, and 2.6% in the lower arch. The need for prosthesis was found in 69.0% and 76.7% of the sample in the upper and lower arches, respectively.

In Table 2 we present the prevalence of the oral condition impact on quality of life, according to the OIDP index. Most individuals (81.9%) reported at least one performance or daily activity affected by oral problems over the last six months prior the survey. The performances “difficulty in eating” (59.5%) and “being ashamed to smile or talk” (59.5%) were the most affected, followed by the performance “feeling uncomfortable when brushing my teeth” (55.2%). The performance with lower prevalence of impact was “the teeth hinder my work” (38.8%).

**Table 2:** Prevalence of oral impact on daily performance (OIDP) of homeless people. Goiânia, state of Goiás, Brazil. (n = 116)

Items	No		Yes		Did not answer	
	n	%	n	%	n	%
1 – Difficulty in eating because of the teeth	44	37.9	69	59.5	3	2.6
2 – Feeling uncomfortable when brushing your teeth	49	42.2	64	55.2	3	2.6
3 – Teeth make you nervous or irritated	53	45.7	58	50.0	5	4.3
4 – Having no fun anymore because of the teeth	65	56.0	47	40.6	4	3.4
5 – Difficulty in speaking because of the teeth	62	53.4	51	44.0	3	2.6
6 – Being ashamed and do not smile anymore	44	37.9	69	59.5	3	2.6
7 – The teeth hinder my work	68	58.6	45	38.8	3	2.6
8 – Slept poorly because of the teeth	65	56.0	48	41.4	3	2.6
Total OIDP*	18	15.5	95	81.9	3	2.6

* Sum of the 8 items.

In Tables 3 and 4 we present the results of bivariate analyses of the association between prevalence of impact (OIDP) and each of the sociodemographic and clinical variables. We found no significant differences concerning sociodemographic characteristics (Table 3).

**Table 3:** Association between sociodemographic variables and oral impact on the daily performance (OIDP) of homeless people. Goiânia, state of Goiás, Brazil. (n = 116)

Variable		Impact (OIDP)				p*
		High		Low		
		n	%	n	%	
Age (years)						0.343
	18–35	28	45.9	28	54.9	
	36–77	33	54.1	23	45.1	
Sex						0.508
	Male	50	82.0	45	86.5	
	Female	11	18.0	7	13.5	
Skin color						0.950
	White	14	23.0	11	22.4	
	Non-white	47	77.0	38	77.6	
Lives with a partner						0.761
	Yes	10	16.4	7	14.3	
	No	51	83.6	42	85.7	
Education level						0.913
	High	19	31.7	16	32.7	
	Low	41	68.3	33	67.3	
Time in the institution (days)						0.172
	1 to 13	22	36.1	24	49.0	
	14 or over	39	63.9	25	51.0	
Time living on the street (years)						0.174
	Up to 1	25	53.2	20	69.0	
	More than 1 year	22	46.8	9	31.0	

* Pearson's Chi-square test.

**Table 4:** Association between oral impact on the daily performance (OIDP) and clinical variables of homeless people. Goiânia, state of Goiás, Brazil. (n = 116)

Variable		Presence of impact (OIDP) – n (%)								
		Item 1	Item 2	Item 3	Item 4	Item 5	Item 6	Item 7	Item 8	High impact
DMFT										
	Low	25 (51.0)	23 (46.9)	23 (46.9)	16 (32.7)	17 (34.7)	30 (61.2)	15 (30.6)	15 (30.6)	23 (46.9)
	High	44 (68.8)	41 (64.1)	35 (56.5)	31 (49.2)	34 (53.1)	39 (60.9)	30 (46.9)	33 (51.6)^a^	38 (59.4)
Decayed teeth										
	No	13 (48.1)	15 (55.6)	12 (46.2)	10 (37.0)	12 (44.4)	13 (48.1)	8 (29.6)	7 (25.9)	13 (48.1)
	Yes	56 (65.1)	49 (57.0)	46 (54.1)	37 (43.5)	39 (45.3)	56 (65.1)	37 (43.0)	41 (47.7)	48 (55.8)
Missing teeth										
	No	65 (62.5)	58 (55.8)	54 (52.4)	43 (41.7)	45 (43.3)	65 (62.5)	42 (40.4)	46 (44.2)	57 (54.8)
	Yes	4 (44.4)	6 (66.7)	4 (50.0)	4 (44.4)	6 (66.7)	4 (44.4)	3 (33.3)	2 (22.2)	4 (44.4)
Using upper prosthesis										
	No	58 (59.2)	54 (55.1)	50 (52.1)	39 (40.2)	42 (42.9)	59 (60.2)	38 (38.8)	40 (40.8)	52 (53.1)
	Yes	11 (73.3)	10 (66.7)	8 (53.3)	8 (53.3)	9 (60.0)	10 (66.7)	7 (46.7)	8 (53.3)	9 (60.0)
Using lower prosthesis										
	No	67 (60.9)	62 (56.4)	57 (52.8)	46 (42.2)	50 (45.5)	67 (60.9)	44 (40.0)	47 (42.7)	60 (54.5)
	Yes	2 (66.7)	2 (66.7)	1 (33.3)	1 (33.3)	1 (33.3)	2 (66.7)	1 (33.3)	1 (33.3)	1 (33.3)
Need for upper prosthesis										
	No	13 (37.1)	14 (40.0)	12 (34.3)	10 (29.4)	11 (31.4)	16 (45.7)	7 (20.0)	8 (22.9)	12 (34.3)
	Yes	56 (71.8)^a^	50 (64.1)^c^	46 (60.5)^c^	37 (47.4)	40 (51.3)	53 (67.9)^c^	38 (48.7)^b^	40 (51.3)^b^	49 (62.8)^b^
Need for lower prosthesis										
	No	13 (48.1)	16 (59.3)	14 (51.9)	10 (38.5)	8 (29.6)	16 (59.3)	10 (37.0)	11 (40.7)	12 (44.4)
	Yes	56 (65.1)	48 (55.8)	44 (52.4)	37 (43.0)	43 (50.0)	53 (61.6)	35 (40.7)	37 (43.0)	49 (57.0)

Pearson's Chi-square test

a p < 0.001

b p < 0.01

c p < 0.05

As for the clinical variables (Table 4), we found significant differences (p < 0.05) between the following variables and OIDP items: DMFT and item 8 (slept poorly); need for upper prosthesis and all OIDP items, except item 4 (have no fun anymore) and item 5 (difficulty in speaking). The only clinical variable associated with the prevalence of total impact (high OIDP) was need for upper prosthesis. We observed a higher proportion of high impact in individuals who require this type of rehabilitation treatment (p = 0.005).

In Table 5 we present the results of the Poisson regression analysis to test the association between prevalence of impact (high OIDP) and need for upper prosthesis. In the unadjusted model, only the “need for upper prosthesis” variable was associated with the outcome (p = 0.015). These variables remained associated even after adjustment for sex, age, and time in the institution. Individuals without need for upper prosthesis had prevalence of high impact on daily performance 55% lower than those in need of this type of prosthesis (p = 0.018).

**Table 5:** Poisson regression results of the association between high oral impact on the daily performance of homeless people and the need for upper prosthesis variable. Goiânia, state of Goiás, Brazil. (n = 110)

Independent variable		Unadjusted		Adjusted	
		PR (95%CI)	p	PR (95%CI)	p
Need for upper prosthesis					
	No	0.55 (0.34–0.89)	0.015	0.55 (0.34–0.90)	0.018
	Yes	1		1	
Age (years)					
	18–35	0.85 (0.60–1.19)	0.345	0.97 (0.70–1.34)	0.849
	36–77	1		1	
Sex					
	Male	0.86 (0.57–1.30)	0.480	0.82 (0.57–1.18)	0.281
	Female	1		1	
Time in the institution (days)					
	1 to 13	0.78 (0.55–1.12)	0.187	0.84 (0.59–1.21)	0.3
	14 or over	1		1	

## DISCUSSION

This is the first study to assess factors associated with the impact of oral health on homeless people in Brazil. The prevalence of negative impact was high in the studied group (81.9%). Among the analyzed clinical variables, only the need for upper prosthesis was associated with the outcome after adjustment for individuals’ sociodemographic characteristics.

Data on the Brazilian population obtained from *Pesquisa Nacional de Saúde Bucal* [National Survey of Oral Health], held in 2010, show lower prevalence of impact (OIDP) in adults and older people (54.9% and 45.3%, respectively)^16^. Considering the precarious living conditions and health care provided by the public services to the homeless population, this result was expected.

In our study, the lack of association of the negative impact with other clinical variables (dental caries, use of prosthesis, and need for lower prosthesis) may be due to the characteristics of the studied group. The homeless situation constantly generates highly-complexity challenges and demands struggle for survival, which compromise the health and other aspects of the lives of individuals, and oral health is just one of them^19^. Thus, unlike what is observed in the overall population, the precarious oral condition of these individuals, such as high percentage of untreated carious teeth and dental loss, may not considerably affect their daily performance. Similarly, the individual sociodemographic factors we studied did not influence on the prevalence of impact.

Tooth loss in the upper arch and no prosthetic replacement, on the other hand, compromise the appearance and quality of life of individuals, since teeth are more exposed in this arch^20^. Although living on the streets, the need for having a good physical appearance, according to conventional aesthetic patterns, seems to be preserved. In addition to appearance, using no prosthesis in the upper arch can affect chewing in a more scathing way than in the lower arch. Due to the difficulty of adjustment of lower dentures, abandoning its use and being used to this situation are frequent, even among the general population.

Previous studies conducted on homeless people from other countries show similar results, in which the clinical variables associated with the negative impact were the number of dental treatments required^8^ and tooth loss^12^. In Hong Kong, however, sociodemographic factors were associated with the impact^10^.

The daily performances most affected in our study were “difficult in eating” and “being ashamed or do not smile anymore,” as well as in the Brazilian population of adults and older people^16^, and reinforce the need for upper prosthesis in the perception of impact in the studied group.

Comparisons with results of previous studies are limited, considering the lack of studies based on the same methodology about homeless people. The only study found in the literature on the quality of life related to oral health of homeless people in Brazil showed prevalence of impact close to our study in men attended by temporary shelter in a municipality of Minas Gerais (78.0%)^14^. In such study^14^, the instrument used to assess the quality of life was the *Oral Health Impact Profile* (OHIP-14) and the most affected dimensions were: psychological discomfort (70% felt embarrassed and tense) and psychological incapacity (54%); clinical conditions and other factors associated with the impact were not investigated.

Studies carried out with homeless population from other countries report prevalence close to our study in England (91%)^12^, Australia (80.4%)^8^, and Hong Kong (88%)^10^. In the United Kingdom, the most frequent impacts were: toothache, discomfort, and chewing difficulty^12^ ^,^ ^13^. As in our study, samples were obtained from institutions that temporarily housed individuals, but using the OHIP-14.

Some limitations should be considered in our study. The size of the study population is small and the results represent homeless people attended by an institution, and cannot be extended to the homeless population of the entire municipality studied. On the other hand, epidemiological surveys on oral health based on the methodological guidelines generally applied in population surveys is unfeasible due to the characteristics of the study population. This difficulty has been observed also in other countries.

Hence, to date, the evidence about the impact of oral health on the quality of life of homeless people is based on temporarily-institutionalized population groups. Considering that the institutions can provide better living conditions to individuals, even if temporarily, and that data were collected during their stay in these places, the perceived impact is expected to be less severe.

Therefore, more studies are needed to broadly understand how oral condition influences on the quality of life of homeless people. Further research should also include individuals who are not attended by institutions housing homeless people, as well as qualitative methods of research to deepen aspects related to the meaning of the oral health in the context of living on the streets.

We conclude that the prevalence of the impact of oral condition on the performance of daily activities of homeless people was high, especially for individuals who require upper prosthesis, regardless of sociodemographic characteristics.

These results strengthen the importance of improving the health care provided to the homeless population and meeting the oral health needs perceived by this extremely disadvantageous group^5^, contributing to a better quality of life and the restoration of their dignity^13^ ^,^ ^21^ ^,^ ^22^. Facilitating the access to treatment and training oral health professionals to provide proper care to these patients are crucial measures^23^.

Whereas the need for prosthesis was the main factor related to the negative impact on the daily performance of homeless people of our study, dental care must go beyond tooth extractions and pain relief, and include dental prosthetic rehabilitation^5^. In addition to existing needs perceived by these people, broader strategies for promoting health care may contribute to the maintenance of oral health in the overall population.
